# Mutant prevention concentration of colistin alone and in combination with rifampicin for multidrug-resistant *Acinetobacter baumannii*

**DOI:** 10.1007/s10096-016-2736-3

**Published:** 2016-08-10

**Authors:** H. Nordqvist, L. E. Nilsson, C. Claesson

**Affiliations:** 1 Department of Infectious Diseases, Linköping University Hospital, Linköping, Sweden; 2 Department of Infectious Diseases, Stockholm South Hospital, Stockholm, Sweden; 3 Clinical Microbiology, Department of Clinical and Experimental Medicine, Linköping University, Linköping, Sweden

## Abstract

Colistin-susceptible isolates of *Acinetobacter baumannii* often contain subpopulations that are resistant to colistin. Monotherapy with colistin can lead to selective growth of these subpopulations and emergence of colistin-resistant strains. Our objectives were to explore the susceptibility pattern of colistin-resistant subpopulations and investigate if combining colistin with a second antibiotic could prevent their selective growth. Four colistin-susceptible clinical isolates of *A. baumannii* and one reference isolate were used. The mutant prevention concentration (MPC) of colistin, i.e. the concentration required to block growth of all single-step-mutant subpopulations, was determined by plating an inoculum of 10^9^ CFU on Mueller Hinton agar (MHA)-plates containing 2-fold dilutions of colistin (0.125–128 mg/L). Susceptibility testing of colistin-resistant subpopulations, obtained in the MPC assay, was performed with Etest. The MPC of colistin, in combination with rifampicin, was determined by plating an inoculum of 10^9^ CFU on MHA-plates containing colistin (0.125–128 mg/L) and fixed concentrations of rifampicin (1.1 mg/L or 4.4 mg/L). The colistin-resistant subpopulations demonstrated increased susceptibility to a number of agents compared to their main populations. These subpopulations were even susceptible to agents that normally are inactive against gram-negative bacteria and all had rifampicin MICs of < 0.002 mg/L. The combination of colistin and rifampicin completely inhibited the growth of all colistin-resistant subpopulations and significantly lowered the MPC of colistin for *A. baumannii*. Combining colistin with rifampicin could be a way to prevent selective growth of colistin-resistant subpopulations of *A. baumannii* and possibly the emergence of colistin-resistant strains.

## Introduction

*Acinetobacter baumannii* has emerged as an important cause of nosocomial infections. Its clinical significance is primarily related to its remarkable ability to upregulate and acquire resistant determinants, making it notoriously difficult to treat [1]. Surveillance studies now show that >80 % of invasive isolates of *A. baumannii* are carbapenem resistant in some European countries [2].

Colistin is an antibiotic with bactericidal effect against gram-negative bacteria. It is often used as a last resort treatment for multi drug-resistant (MDR) gram-negative bacteria, including carbapenem-resistant *A. baumannii* [3]. Unfortunately, colistin-resistant strains of *A. baumannii* have emerged [4, 5]. As a consequence we now face the risk of infections caused by *A. baumannii* that cannot be treated with antimicrobials.

Heteroresistance to colistin due to resistant subpopulations is a well-described phenomenon among *A. baumannii* [4]. Colistin-resistant strains can develop if the colistin concentration is sufficient to prevent growth of the susceptible main population but not the resistant subpopulations [6]. The concentration required to prevent such selective growth is called the mutant prevention concentration (MPC). The MPC is defined as the concentration required to block growth of all single-step-mutant subpopulations [7, 8].

The MPC of colistin in vitro for *A. baumannii* has been shown to be so high that it cannot be exceeded in vivo. These results suggest that monotherapy with colistin will inevitably lead to selective growth of colistin-resistant subpopulations. The MPC of colistin against *A. baumannii* was lowered when colistin was combined with levofloxacin or tobramycin. It was however still well beyond clinically achievable concentrations [9, 10]. The results from one study implicate that other agents, for example, rifampicin and meropenem, may be more effective in lowering the MPC [11].

Our first objective was to explore the susceptibility pattern of colistin-resistant subpopulations of *A. baumannii*. Our second objective was to investigate if we could prevent their selective growth by combining colistin with a second antibiotic that the colistin-resistant subpopulations were susceptible to.

## Material and methods

### Characterization of bacterial isolates

Four clinical isolates (AB1-AB4) of *A. baumannii* isolated from four different patients during 2013 at the Department of Clinical Microbiology, Linköping University Hospital (Linköping, Sweden) were included. Species identification was done with MaldiTof (Bruker Daltonics Scandinavia AB, Solna Sweden). Pulse-field gel electrophoresis (PFGE) and determination of the resistance genes present in AB1-AB4 were performed at the Public Health Agency of Sweden (Solna, Sweden). According to PFGE the four isolates represented four different clones. The following carbapenemase genes were identified: OXA 23 (AB1-AB3), OXA 51 (AB1-AB4), OXA 58 (AB4) and NDM (AB1). All clinical isolates were defined as MDR according to proposed nomenclature [12], but classified as susceptible to colistin (S ≤ 2 mg/L) [13]. *A. baumannii* CCUG 19096 (AB5), *Pseudomonas aeruginosa* ATCC 27853 and *Escherichia coli* ATCC 25922, obtained from the Culture Collection, Göteborg University, Sweden, were used as type strains.

### MIC of colistin determined with Etest, broth dilution and agar dilution

Colistin MIC was determined for isolates AB1-5 with Etest (bioMérieux, Marcy l’Etoile, France), broth dilution and agar dilution. Colistin Etest was applied on Mueller-Hinton Agar (MHA) (Becton Dickinson, Franklin Lakes, NJ, USA) inoculated with a bacterial suspension of 0.5 McFarland in 0.85 % NaCl and was read after 24 h incubation at 36 °C. Broth dilution MIC of colistin sulphate (Sigma-Aldrich corp., St Louis, MO, USA) was determined with 2-fold dilutions (0.016–256 mg/L) in glass tubes containing 1 mL Mueller-Hinton Broth (MHB) (Becton Dickinson), with an inoculum of approximately 1 × 10^5^ CFU/mL. Broth dilution MIC was defined as the lowest concentration inhibiting visible growth after 24 h of incubation at 36 °C. Agar dilution MIC was determined with MHA (Becton Dickinson) plates with 2-fold dilutions of colistin sulphate (Sigma Aldrich), ranging from 0.08 to 2.0 mg/L. A bacterial suspension of approximately 10^5^ CFU/mL, determined by viable count, was prepared by dilution with NaCl 0.85 % of an overnight culture of 3 mL MHB (Becton Dickinson). Plates were spot inoculated, with two spots and 10^4^ CFU in each spot. The plates were incubated overnight at 36 °C. Agar-MIC was defined as the lowest concentration that inhibited visible growth. All tests were performed in duplicates to ensure reproducibility.

### MPC of colistin determined with agar dilution

The MPC was determined with a method described by Cai et al. [9], but with modifications. Isolates AB1-5 were grown overnight on Columbia agar (Acumedia, Lansing, MI, USA) with 5 % defibrinated horse blood (Håtuna, Uppsala, Sweden) at 36 °C. Glass tubes containing 2 mL MHB (Becton Dickinson) were inoculated with bacteria from the Columbia agar and incubated overnight at 36 °C with shaking, reaching a bacterial concentration of 10^9^ CFU/mL, determined by viable count. Aliquots of 1 mL were plated on MHA-plates (Becton Dickinson) containing colistin sulphate (Sigma Aldrich) in 2-fold dilutions (0.125–128 mg/L). The MPC was defined as the lowest concentration of colistin that inhibited all visible growth of 10^9^ CFU after 48 h of incubation [7]. Colonies were isolated from the plates with the highest and the second highest colistin concentration with growth of bacteria. MaldiTof (Bruker) was used to determine that the colonies were *A. baumannii*, and Etest (bioMérieux) was used to determine their susceptibility to colistin. The tests were performed in duplicates to ensure reproducibility.

### MIC of the main populations (AB1-5) and the colistin-resistant subpopulations determined with Etest

The main populations of the isolates AB1-5 and the colistin-resistant subpopulations isolated from the colistin plates in the MPC assay were studied further. The MIC of colistin, vancomycin, rifampicin, meropenem, linezolid, ampicillin/sulbactam, tigecycline, ciprofloxacin and tobramycin was determined with Etest (bioMérieux) on MHA-plates (Becton Dickinson) inoculated with a bacterial suspension adjusted to 0.5 McFarland in 0.85 % NaCl. The MIC was read after 24 h of incubation at 36 °C for vancomycin and colistin and after 18 h for the remaining agents.

### MPC of colistin in combination with rifampicin determined with agar dilution

Bacterial suspensions with a concentration of 10^9^ CFU/mL were generated for isolate AB1-AB5 as described above in the method for the MPC of colistin determined with agar dilution. Aliquots of 1 ml were plated on MHA-plates (Becton Dickinson) containing colistin sulphate (Sigma Aldrich) 0.125–128 mg/L (using 2-fold dilutions) in combination with fixed concentrations of rifampicin (Sigma Aldrich) 1.1 or 4.4 mg/L. We choose concentrations of rifampicin that we considered clinically relevant. Free unbound concentration of 1.1 mg/L can readily be achieved with per oral administration of 600 mg rifampicin and 4.4 mg/L with 900 mg [14, 15]. The tests were performed in duplicates to ensure reproducibility. The MPC was defined as the lowest concentration of colistin that inhibited all visible growth of an inoculum of 10^9^ CFU after 48 h of incubation at 36 °C [7]. Colonies were isolated from the plates with the highest and the second highest colistin concentration with growth of bacteria. MaldiTof (Bruker) was used to determine that the colonies were *A. baumannii* and Etest (bioMérieux) to determine their susceptibility to colistin and rifampicin.

## Results

### Colistin MIC

The MIC of colistin for the main population determined with Etest, agar dilution and broth dilution were 0.125, 0.25–0.5 and 0.50–1 mg/L, respectively (Table 1).

**Table 1:** MIC determination of colistin with Etest, agar and broth dilution assay

Isolate	Etest MIC (mg/L)	Agar dilution MIC (mg/L)	Broth dilution MIC (mg/L)
AB1	0.125	0.5	1
AB2	0.125	0.5	1
AB3	0.125	0.25	1
AB4	0.125	0.25	0.5
AB5	0.125	0.5	1

### MPC of colistin alone

**Fig. 1:**
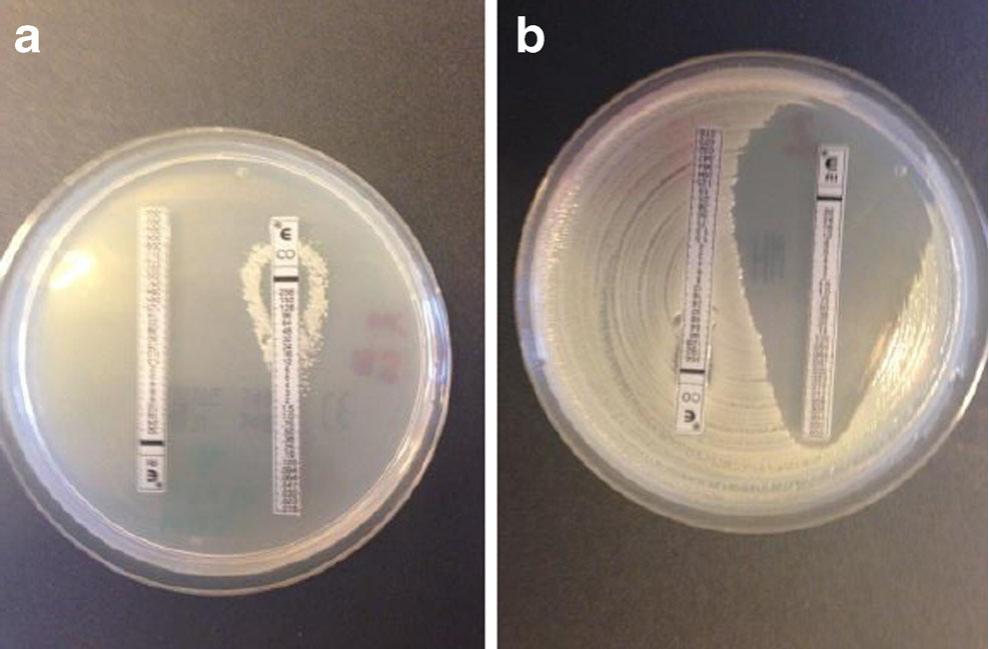
MIC for rifampicin and colistin after overnight incubation on MHA. **a** Colistin-dependent growth—colonies growing only along the colistin Etest strip. **b** Colistin-resistant subpopulation with MIC for rifampicin < 0.002 mg/L

The MPC was 64 mg/L for all isolates except AB3 that displayed an MPC-value of 16–32 mg/L (Table 2). Colistin-resistant subpopulations were recovered from MHA-plates containing 16–32 mg/L colistin for all tested isolates in the frequency of 1 of 10^7^–10^9^, except for AB3 from which no colistin-resistant subpopulations were recovered. Subpopulations displaying colistin-dependent growth were also recovered from colistin-containing plates for isolates AB1 and AB2 (Fig. 1a). Colistin-susceptible cells were detected on plates containing colistin 8–32 mg/L in all isolates. They were most often found co-existing with colistin-resistant subpopulations, but not always.

**Table 2:** Mutant prevention concentration (MPC) of colistin alone and in combination with rifampicin 1.1 mg/L or 4.4 mg/L

Isolate	MPC (mg/L)		
	Colistin alone	Colistin + 1.1 mg/L rifampicin	Colistin + 4.4 mg/L rifampicin
AB1	64	8–16	8–16
AB2	64	16	8–16
AB3	16–32	16–32	32
AB4	64	8	8
AB5	64	8–16	8–16

### Antibiograms for main populations and colistin-resistant subpopulations

MIC values with Etest were determined for the main populations and the colistin-resistant subpopulations (Table 3). The main population of four isolates (AB1-4) were MDR [12] with high MIC values for all antimicrobial agents tested, except for colistin. The main population of one isolate displayed high-level resistance to rifampicin >32 mg/L (AB3) while the other isolates' main populations had MIC values of 4–8 mg/L. The colistin-resistant subpopulations clearly displayed increased susceptibility to rifampicin, vancomycin and meropenem compared to the main populations. The rifampicin MICs were much lower (<0.002 mg/L) for all colistin-resistant subpopulations compared to the main populations (Fig. 1b).

**Table 3:** Etest MIC of main population and colistin-resistant subpopulations of isolates AB1-AB5

Isolate	MIC (mg/L) of main population/colistin-resistant subpopulation							
	Colistin	Rifampicin	Vancomycin	Linezolid	Meropenem	Ciprofloxacin	Tigecycline	Tobramycin
AB1	0.125/128	8/<0.002	>256/0.125	>256/32	>32/0.064–0.25	>32/15– > 32	4/0.5	12/0.5–2
AB2	0.125/12–128	8/<0.002	>256/0.25–1	>256/32–128	>32/0.125–0.5	>32/≥32	4/0.5–1	2/0.5–2
AB3	0.125/–	>32/–	>256/–	>256/–	>32/–	>32/–	2/–	>256/–
AB4	0.125/>256	8/<0.002	>256/2	>256/32	4/0.064	>32/>32	4/1	2/1
AB5	0.125/16–32	4/<0.002	>256/0.125	>256/32	1/0.016	>32/0.032	2/0.064	4/0.064

For AB3 only Etest MIC of main population is presented, since no colistin-resistant subpopulations were detected in this isolate

### MPC of colistin in combination with rifampicin

We chose to combine colistin with rifampicin because of the results in the antibiogram study described above. The MPC-values of colistin when combined with rifampicin at fixed concentrations of 1.1 mg/L or 4.4 mg/L are reported in Table 2. The MPC was lowered from 64 mg/L to 8–16 mg/L for the isolates AB1, AB2, AB4 and AB5 when colistin was combined with rifampicin 1.1 mg/L or 4.4 mg/L. The MPC was not lowered for the AB3 isolate. No difference regarding the MPC could be seen when comparing plates containing rifampicin 1.1 mg/L and rifampicin 4.4 mg/L. Interestingly, no colistin-resistant subpopulations were detected on any of the MHA plates containing both colistin and rifampicin 1.1 mg/L or 4.4 mg/L. We detected colistin-susceptible cells with high-level resistance against rifampicin on MHA-plates containing both colistin and rifampicin in all isolates. No difference could be seen when comparing plates containing rifampicin 1.1 mg/L and 4.4 mg/L.

## Discussion

Colistin-resistant strains of *A. baumannii* can develop during colistin therapy if colistin-resistant subpopulations grow selectively [16–19]. We hypothesized that such growth could be prevented if colistin was combined with an agent effective against these subpopulations. The colistin-resistant subpopulations were extremely susceptible to rifampicin (MIC < 0.002 mg/L) and the combination of colistin and rifampicin completely inhibited the growth of all colistin-resistant subpopulations and significantly lowered the MPC of colistin.

The colistin-resistant subpopulations demonstrated increased susceptibility to a number of agents compared to their main population. They were even susceptible to rifampicin and vancomycin, agents that normally are inactive against gram-negative bacteria. These findings are likely due to increased outer membrane permeability, allowing greater access to target sites for these agents. One study reported similar results [11]. Meropenem and especially rifampicin demonstrated the greatest potential to prevent selective growth, with MICs for the colistin-resistant subpopulations of 0.016–0.5 mg/L and < 0.002 mg/L, respectively. Rifampicin has the advantage of not being nephrotoxic, but has the disadvantages of possible hepatotoxicity and drug interactions [20]. The broad spectrum of meropenem could be of benefit since many patients infected with *A. baumannii* are co-infected [20], while disadvantages are possible nephrotoxicity when it is combined with colistin [21] and the risk of an increase in carbapenem resistance if carbapenem consumption is increased [22].

We chose to combine colistin with rifampicin because of the colistin-resistant subpopulations extreme susceptibility to this agent. To our knowledge no previous study has investigated the effect on the MPC of colistin for *A. baumannii* when rifampicin is added. In our study the MPC of colistin was lowered ≥ 2 dilution steps in all heteroresistant isolates when rifampicin was added in clinically achievable concentrations. No colistin-resistant subpopulations were recovered from any MHA-plates containing both colistin and rifampicin. These results show that rifampicin can prevent selective growth of colistin-resistant subpopulations in vitro. Our results are supported by other in vitro studies [19, 23]. Most in vitro studies have shown 100 % synergy for the colistin-rifampicin combination against MDR *A. baumannii* with low to intermediate rifampicin resistance (MIC ≤ 16 mg/L) [24, 25]. It is possible that a synergistic effect contributed to the lowered MPC seen in our heteroresistant isolates.

Evidence that rifampicin prevents the development of colistin-resistance in vivo is still lacking, although a colistin-resistant strain of *A. baumannii* has successfully been treated with rifampicin [19]. We have found only one in vivo study that has investigated this issue (secondary outcome). No difference regarding colistin-resistance development could be seen when comparing colistin vs. colistin and rifampicin, since no development of colistin-resistance could be detected in any patient [20]. While our study and many in vitro studies [9, 10, 16–18] indicate a very high risk of development of colistin-resistance with colistin alone, in vivo studies show conflicting data [19, 20, 26]. This discrepancy in vivo could be due to different length of follow up [20, 26], the wide use of concomitant antibiotics in vivo [20], colistin dosage [20], reduced ability for the colistin-resistant organisms to survive and replicate in vivo [27] or overestimation of the MPC in vitro [7]. We believe that the MPC of colistin for *A. baumannii* is overestimated in vitro due to the inoculum effect for colistin [28]. No colistin-susceptible cells could be detected on plates containing ≥ 0.5 mg/L colistin when using an inoculum of 10^5^ CFU, but up to 32 mg/L with 10^9^ CFU.

No colistin-resistant subpopulations were detected in AB3 and the MPC of colistin alone was also 1–2 dilutions steps lower for this isolate. The MPC of colistin was not affected for AB3 when rifampicin was added. This was expected due to lack of colistin-resistant subpopulations and the high-grade rifampicin resistance (MIC > 16 mg/L) displayed by AB3. High-grade rifampicin resistance is mediated by rpoB-mutations. No additive or synergistic effect is expected for the colistin-rifampicin combination against isolates carrying this mutation [25]. To our knowledge no correlation has been found between rpoB-mutation and lack of colistin-resistant subpopulations. We therefore believe that the combination of findings in AB3 were a mere coincidence.

We did not detect any colistin-resistant cells with rifampicin resistance, but development of resistance due to rpoB mutations is always a concern when using rifampicin. This mutation can occur spontaneously or upon rifampicin exposure and colistin-susceptible *A. baumannii*, like AB3, readily acquire it [20]. Compared to the main populations, colistin-resistant subpopulations exist in substantially lower numbers [8, 19] and are significantly more susceptible to rifampicin [11]. It is therefore less likely that the colistin-resistant subpopulation would mutate in the rpoB gene spontaneously or upon rifampicin exposure compared to the colistin-susceptible main population.

Colistin susceptibility testing is problematic and disconcordant results for different susceptibility testing methods are a well-known problem [29–32]. The categorical agreement was 100 % for Etest, agar and broth dilution in our study. Only Etest was used for colonies isolated in the MPC-study to differentiate between colistin susceptible and resistant cells. This method was chosen for practical reasons and because very good performance has been demonstrated with Etest for colistin [29, 30]. However, a risk of Etest falsely classifying isolates as colistin susceptible has been demonstrated. We had no Etest results in the range (0.5–2.0 mg/L) for which the absolute majority of these erroneous results have been demonstrated [31, 32]. We therefore assess that the likelihood for false susceptible results in our study is low.

One interesting finding in our study was subpopulations that exhibited a colistin-dependent growth pattern. This phenomenon has previously been described for colistin and *A. baumannii*. The authors suggest that one possible mechanism for colistin-dependent growth may be a mutation of lipid A, which results in a defective cell membrane and osmotic trauma in the absence of colistin [33]. The clinical importance of these subpopulations is yet to be determined.

In conclusion, our results indicate a risk of selective growth of colistin-resistant subpopulations with colistin monotherapy against *A. baumannii* and that combining colistin with rifampicin could be a way to prevent this occurrence.
